# S123. TREATMENT RESISTANT SCHIZOPHRENIA AND GYRIFICATION-BASED CONNECTOME

**DOI:** 10.1093/schbul/sby018.910

**Published:** 2018-04-01

**Authors:** Olesya Ajnakina, Tushar Das, John Lally, Marta Di Forti, Robin Murray, Lena Palaniyappan

**Affiliations:** 1 Institute of Psychiatry, King’s College London; 2 University of Western Ontario

## Abstract

**Background:**

Treatment-resistant schizophrenia (TRS) is a major cause of disability and functional impairment worldwide. Approximately 30% of patients with schizophrenia will develop TRS at some point during their illness course. Despite the staggering financial and emotional costs associated with TRS, this severe disorder is poorly understood. The pathophysiological basis of TRS is posited in part to have neurodevelopmental roots. If early brain development (<2 years of age) influences TRS, then cortical gyrification, which is often complete by 2 years of life, could be abnormal in TRS when compared to non-TRS subjects. Subtle but diffuse pathological changes that occur during early development are postulated to disrupt the maturational relationship (covariance) among brain regions, even if no localised morphological changes are seen in adult life. The disrupted structural covariance resulting from diffuse developmental dyscoordination in early life can be quantified using gyrification-based connectomes obtained using graph theory. We applied this method to baseline MRI data collected during first contact with mental health services for psychosis to predict the emergence of TRS in the next 5 years.

**Methods:**

70 patients with first episode schizophrenia spectrum disorder who presented to mental health services between 2005 and 2010 were followed up for 5 years using electronic case notes. Psychopathology was assessed at baseline with the Positive and Negative Syndrome Scale (PANSS) and symptom dimensions were derived using Wallwork’s model. TRS was defined according to Health and Clinical Excellence guidelines. Structural MRI images were obtained at baseline, with minimal exposure to antipsychotics (<3 months). Local gyrification indices were computed using Schaer’s method for 68 contiguous cortical regions (34 in each hemisphere) using Freesurfer’s Desikan atlas. After adjusting for age, gender and intracranial volume, group-based structural covariance was estimated (68x68 correlation indices) and each subject’s contribution to the covariance was quantified using a jack-knife procedure, providing one distance matrix for each subject. These matrices were used to construct distance-based gyrification connectomes using Graph Analysis Toolbox. We used a functional data analysis approach across a range of cost-thresholds to reduce multiple testing when comparing TRS and non-TRS groups.

**Results:**

17 (24.3%) of patients with first episode schizophrenia spectrum disorder met criteria for TRS at the end of the 5 years of follow up; 53 (75.7%) were non-TRS. TRS subjects had a significant reduction in small-worldness compared to non-TRS group (Hedges’s g=2.09, p<0.001) and reduced clustering coefficient (Hedges’s g=1.07, p<0.001) with increased path length (Hedges’s g=-2.17, p<0.001).The positive symptoms were positively correlated (after adjusting for age, gender and TRS status) with higher small-worldness (r=0.414, p=0.001) suggesting that a predominantly hyperdopaminergic status that induces positive symptoms may relate to preserved small-worldness seen in non-TRS individuals, while subtle developmental changes resulting in reduced small-worldness may underlie TRS.

**Discussion:**

These changes suggest that in the presence of TRS, the cortex-wide covariance in folding patterns become less organized, with reduced regional segregation as well as reduced overall integration of the morphological connectome. Such an effect may result from weakening of the tensions that arise from inter-regional connectivity in the neonatal brain. The emergence of TRS may be characterised by a neurodevelopmentally driven abnormality in structural organisation of the human cortex in those who develop schizophrenia.

